# A novel recombinant javanicin with dual antifungal and anti-proliferative activities

**DOI:** 10.1038/s41598-019-55044-7

**Published:** 2019-12-05

**Authors:** Santhasiri Orrapin, Amornrat Intorasoot, Sittiruk Roytrakul, Nathupakorn Dechsupa, Jiraporn Kantapan, Yanika Onphat, Chutima Srimek, Chayada Sitthidet Tharinjaroen, Usanee Anukool, Bordin Butr-Indr, Ponrut Phunpae, Sorasak Intorasoot

**Affiliations:** 1 Division of Clinical Microbiology, Department of Medical Technology, Faculty of Associated Medical Sciences, Chiang Mai University, Chiang Mai, 50200 Thailand; 2 Department of Microbiology, Faculty of Medicine, Chiang Mai University, Chiang Mai, 50200 Thailand; 3 Proteomics Research Laboratory, National Center for Genetic Engineering and Biotechnology (BIOTEC), Thailand Science Park, Pathum Thani, 12120 Thailand; 4 Department of Radiologic Technology, Faculty of Associated Medical Sciences, Chiang Mai University, Chiang Mai, 50200 Thailand; 5 Infectious Diseases Research Unit (IDRU), Faculty of Associated Medical Sciences, Chiang Mai University, Chiang Mai, 50200 Thailand

**Keywords:** Applied microbiology, Antimicrobial resistance

## Abstract

Resistance to common drugs by microorganisms and cancers has become a major issue in modern healthcare, increasing the number of deaths worldwide. Novel therapeutic agents with a higher efficiency and less side effects for the treatment of certain diseases are urgently needed. Plant defensins have an integral role in a hosts’ immune system and are attractive candidates for combatting drug-resistant microorganisms. Interestingly, some of these defensins also showed great potential due to their cytotoxic activity toward cancer cells. In this study, a defensin encoding gene was isolated from five legume seeds using 3′ rapid amplification of cDNA ends (3′ RACE) with degenerate primers and cDNA cloning strategies. Bioinformatic tools were used for *in silico* identification and the characterization of new sequences. To study the functional characteristics of these unique defensins, the gene encoded for *Sesbania javanica* defensin, designated as javanicin, was cloned into pTXB-1 plasmid and expressed in the *Escherichia coli* Origami 2 (DE3) strain. Under optimized conditions, a 34-kDa javanicin-intein fusion protein was expressed and approximately 2.5–3.5 mg/L of soluble recombinant javanicin was successfully extracted with over 90% purity. Recombinant javanicin displayed antifungal properties against human pathogenic fungi, including resistant strains, as well as cytotoxic activities toward the human breast cancer cell lines, MCF-7 & MDA-MB-231. Recombinant javanicin holds great promise as a novel therapeutic agent for further medical applications.

## Introduction

The emergence of drug resistant microorganisms and cancers has become a serious problem worldwide, between the two they are a leading cause of disease and mortality in human populations. Overuse of pharmaceutical drugs, inappropriate prescribing of medications and prolonged therapies are all associated with the increased number of drug-resistant microorganisms^1^. Currently, the World Health Organization has revealed that cancers are the second leading cause of death globally^2^. Several factors can cause abnormal and uncontrolled cell growth (cancer) including exposure to radiation, certain chemicals, aging and infections from some bacteria and viruses^3,4^. Patients with cancer and/or participating in anticancer therapies are at particular risk of being infected by opportunistic pathogens^5^. Moreover, multi-drug resistant (MDR) microbial infections in cancer patients may lead to complications in their treatment and possibly chronic illness. Recently, the death rates and medical complications for people suffering from cancer and infected with MDR microorganisms are increasing annually^5^. Unique therapeutic agents with higher efficiency and decreased side effects for the treatment of these hard to cure diseases are urgently needed. Regarding their multifaceted effects, antimicrobial peptides (AMPs), a natural host defense peptide, are one of the more attractive agents for the treatment of these issues, which are not only capable combatting both drug-sensitive and drug-resistant microbial pathogens but also exhibit antiviral and anticancer activities. Furthermore, AMPs also induce modulatory functions in the innate immune response^6^. Although these peptides exact mode of action towards cancer cells is still unclear, several modes of function on microorganisms have previously been reported, one particular activity, membrane disruption demonstrates a major mechanism for microbial growth inhibition. The structures and activities of AMPs are well-documented and largely dependent on their physicochemical properties including cationicity, hydrophobicity, and amphipathicity^7^. Unlike conventional antibiotics, AMPs represent broad-spectrum antimicrobial activity with a low propensity to induce drug resistance, even after long-term treatment.

AMPs are produced by a wide variety of organisms ranging from bacteria to mammals. Among these, plants have attracted the most attention as an accessible source for antimicrobial compounds^8^. Many plant AMPs have previously been documented and classified based on some common features such as small cationic, amphipathic and conserved cysteine residues that conform their structures by pairing to disulfide bridges. Defensins are one of the earliest innate immune systems to show up in plants and are expressed either in storage organs or by inducible production upon pathogenic attack^9^. Plant defensins have been isolated from various plant organs however they are mostly found in seeds^10^. The peptide generally has 45–54 amino acid residues with a three-dimensional αβ-motif folding pattern stabilized by 4–5 disulfide bonds^11^. To date, numerous biological functions for plant defensins have been identified and published including antibacterial, antifungal, insecticidal and cytotoxic effects^12^. Notably, most defensins demonstrated single actions on either antimicrobial or cytotoxic levels, therefore, some of these exhibited multiple bioactivities. The plant defensins with dual antimicrobial and anti-proliferative properties NaD1 and PvD1, isolated from *Nicotiana alata* and *Phaseolus vulgaris*, respectively, have been studied and published^13,14^. While there are similarities between amino acid sequences including comparible secondary structures and antimicrobial activities have been observed, the actual mode of actions of each plant defensin, including their target molecules and subcellular localization, show that they are extremely diverse^15,16^.

Large-scale AMP production with high purity is currently in high demand for pharmaceutical applications. Several strategies have been employed for obtaining large amounts of AMPs. Although AMPs have been directly isolated from crude plant extracts, multi-step peptide purification, stability and low recovery yields are some of the limitations for this process. While chemical peptide synthesis is favorable for AMP production, due to its high purity, the cost is a major limitation, particularly in long peptide synthesis^17^. Due to the cost-effectiveness and ease of scalability, recombinant protein expression in prokaryotes is a preferable alternative in the production of active AMPs. Recently recombinant AMP expressions have become more reliable with higher yields of purified peptides from the bacteria, overall the process has greatly improved. Any problems arising due to the toxicity of AMPs on their expression host can be overcome with the use of fusion partners. In addition, fusion partners are also capable of protecting AMPs from proteolytic cleavage, improving the purification process, increasing the expression efficiency and improving the solubility of recombinant peptides^18^.

The objectives of this study were the isolation, identification, recombinant production and functional characterization of new plant defensins in the Fabaceae plant family. The total RNAs from five tropical legume seeds, including *V. mungo*, *C. juncea, L. purpureus*, *S. javanica* and *C. gladiata*, were isolated, then reverse transcribed to cDNA and amplified through a 3′RACE using specific degenerate primers designed from conserved sequences corresponding to legume defensins. General bioinformatic tools were utilized to identify the obtained DNA sequences. One candidate gene encoded for javanicin, a new plant defensin from *S. javanica*, was employed for cloning and expression in *E. coli* using the intein-mediated protein expression system. A recombinant javanicin antimicrobial peptide was produced and purified for cytotoxic analysis and antimicrobial effects against drug-sensitive and drug-resistant microorganisms.

## Results

### Isolation, identification and *in silico* analysis of gene encoding for potential plant defensins

**Figure 1:**
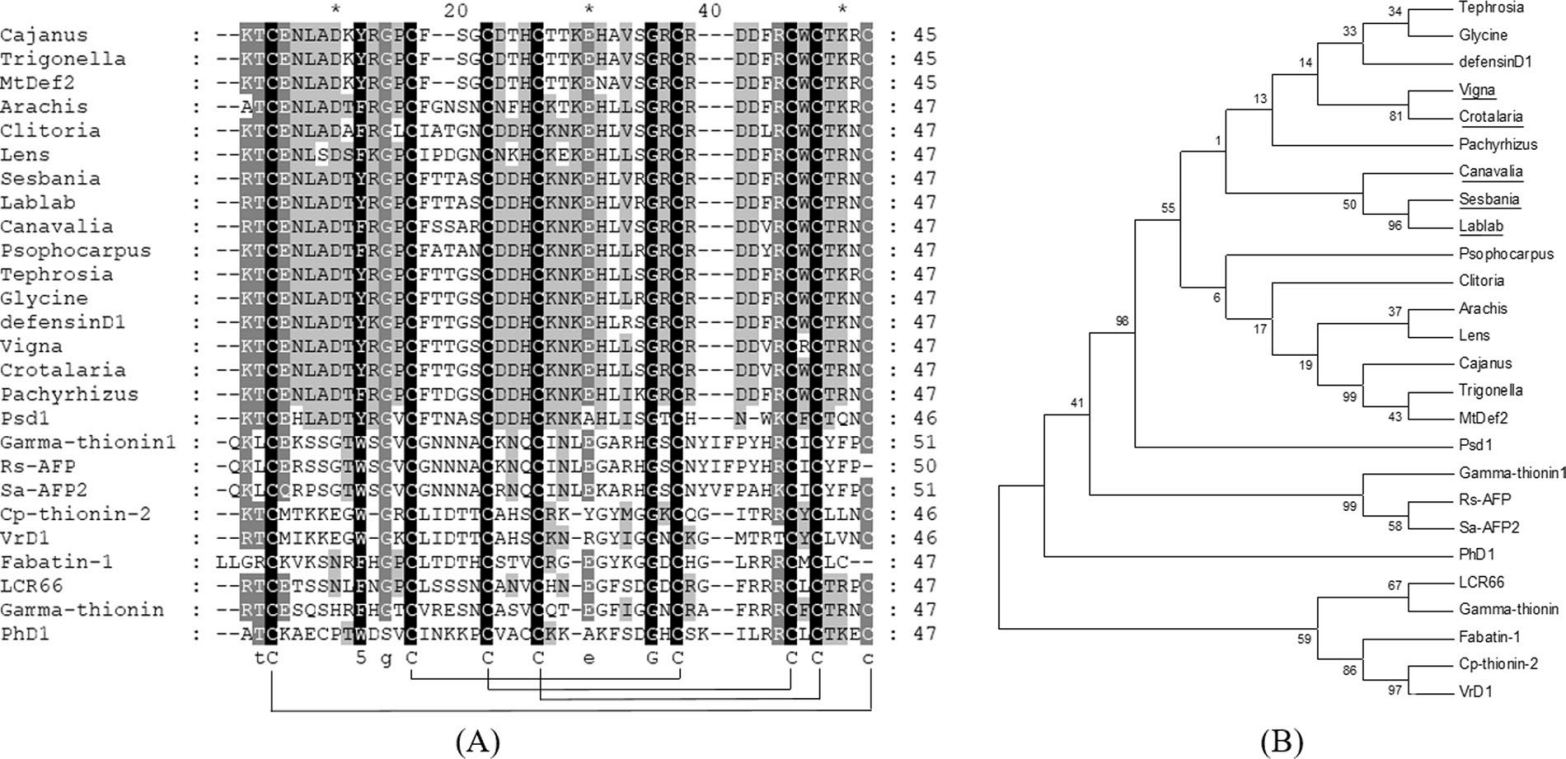
The amino acid sequence alignment and phylogenetic analysis of plant defensins. Deduced amino acid sequence of five legume defensins including *V. mungo*, *C. juncea*, *L. purpureus, S. javanica* and *C. gladiata* identified in this study were aligned with other known defensins from the Fabaceae family and other clusters including the Brassicaceae and Solanaceae families (**A**). The phylogenetic tree was created for evolutionary correlation of novel (underlined) and other known plant defensins (**B**). Tephrosia, *Tephrosia villosa*; Glycine, *Glycine max*; defensinD1, *Phaseolus vulgaris*; Vigna, *V. mungo*; Crotalaria, *C. juncea*; Pachyrhizus, *Pachyrhizus erosus*; Canavalia, *C. gladiata*; Sesbania, *S. javanica*; Lablab, *L. purpureus*; Psophocarpus, *Psophocarpus tetragonolobus*; Clitoria, *Clitoria ternatea*; Arachis, *Arachis diogoi*; Lens, *Lens culinaris* subsp. *culinaris*; Cajanus, *Cajanus cajan*; Trigonella, *Trigonella foenum-graecum*; MtDef2, *Medicago truncatula*; Psd1, *Pisum sativum*; Gamma-thionin1, *Wasabia japonica*; Rs-AFP, *Raphanus sativus*; Sa-AFP2, *Sinapis alba*; PhD1, *Petunia hybrida*; LCR66, *Arabidopsis thaliana*; Gamma-thionin, *Petunia integrifolia*; Fabatin-1, *Vicia faba*; Cp-thionin-2, *Vigna unguiculata*; VrD1, *Vigna radiate*.

A full length defensin gene from legume seeds was successfully amplified by 3′ RACE using degenerate primers corresponding to a Fabaceae plant defensin. The PCR product was purified, ligated and transformed into *E. coli* TOP 10 F. Direct sequencing was performed for a complete nucleotide sequence analysis. The nucleotide and deduced amino acid sequences of these unique plant defensins from the seeds of *V. mungo*, *C. juncea*, *L. purpureus, S. javanica* and *C. gladiata* were recorded in GenBank accession No. MH045506-MH045510, respectively. Several bioinformatic tools were employed to predict the physicochemical properties of plant defensin in this study. Initially, a nucleotide sequence was translated to an amino acid sequence. The results indicated that these defensin antimicrobial peptides were highly conserved with a 75-amino acids pro-peptide consisting of a 28 amino acids signal sequence analyzed by SignalP 4.1 and the C-terminal 47 residues mature peptide. The predicted molecular mass of these mature peptides ranged from 5.38–5.56 kDa with a net positive charge of +1 to +2 and an isoelectric point (pI) of approximately 7.72–8.22. The CAMP software was utilized for antimicrobial peptide prediction through the three most common algorithms. These included Support Vector Machine (SVM), Random Forest (RF) and Discriminant Analysis (DA) and the results gave high probability scores, indicating that these unique plant peptides had a high likelihood of being antimicrobial peptides. For evolution analysis, the deduced amino acid sequences of new plant defensins were subsequently aligned with other known plant defensins using the Clustal X 2.1 program and displayed using GeneDoc 2.7 public software. The results of multiple sequence alignments are shown in Fig. 1A. A phylogenetic tree was generated with the Neighbor Joining (NJ) method, created using MEGA 6 and the branches were examined with 1000 bootstrap replicates. The results from the phylogenetic analysis indicated that these new plant defensins were highly conserved with eight conserved cysteine residues as previously reported^19^. The result of phylogenetic analysis is shown in Fig. 1B.

### Generation of pTxB1-Javanicin plasmid

**Figure 2:**
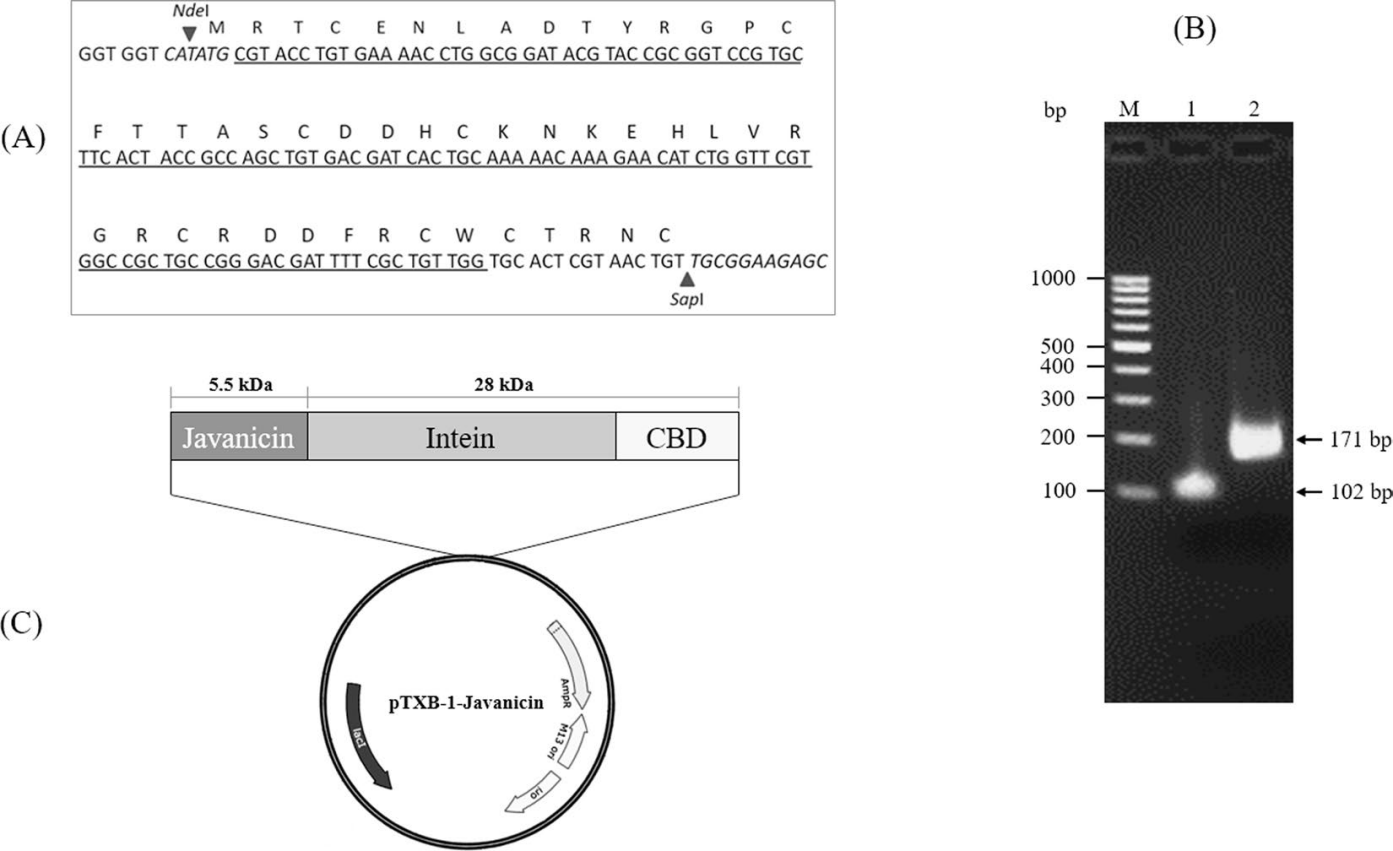
Schematic representation of the construction of recombinant javanicin. The *E. coli* codon usage nucleotide encoded for mature javanicin was constructed by franking with *Nde*I and *Sap*I cleavage sites at 5′ and 3′ end, respectively (**A**). The gene construct was amplified by SOE-PCR (**B**). After restriction of enzyme digestion, the javanicin gene was ligated into the N-terminal end of intein-CBD fusion partner of pTXB-1 plasmid (**C**). Lane M, 100-bp DNA ladder marker; Lane 1, the 1^st^ round of javanicin gene construct (102 bp) amplified using P1 & P2 overlapped primers and Lane 2, the full length javanicin gene construct (171 bp) amplified using P3 & P4 primers with *Nde*I and *Sap*I cleavage sites at the 5′ and 3′ end and the 1^st^ round amplified product was utilized as template for the full length gene amplification.

To study the functional characteristics of these new defensins, one of these plants, *S. javanica*, was selected as a candidate for further characterization. General information for the *S. javanica* defensin was analyzed and the results indicated that the predicted molecular mass of the peptide was 5.56 kDa with a net positive charge of +2 and an isoelectric point (pI) of 8.21. A 171-bp fragment encoded for a mature javanicin gene flanked by *Nde*I and *Sap*I cleavage sites was successfully amplified according to *E. coli* codon usage using a spliced overlap extension-polymerase chain reaction (SOE-PCR) (Figs. 2A,B). After restriction enzyme digestion, the target gene was ligated into a linearized pTXB-1 expression vector (Fig. 2C) and transformed into *E. coli* origami 2 (DE3). Bacteria harboring recombinant plasmids were selected by colony-PCR. The nucleotide sequence of javanicin-intein-chitin-binding domain (CBD) was verified to be correct by direct sequencing and theoretically an optimized codon (data not shown).

### Expression and purification of recombinant javanicin

**Figure 3:**
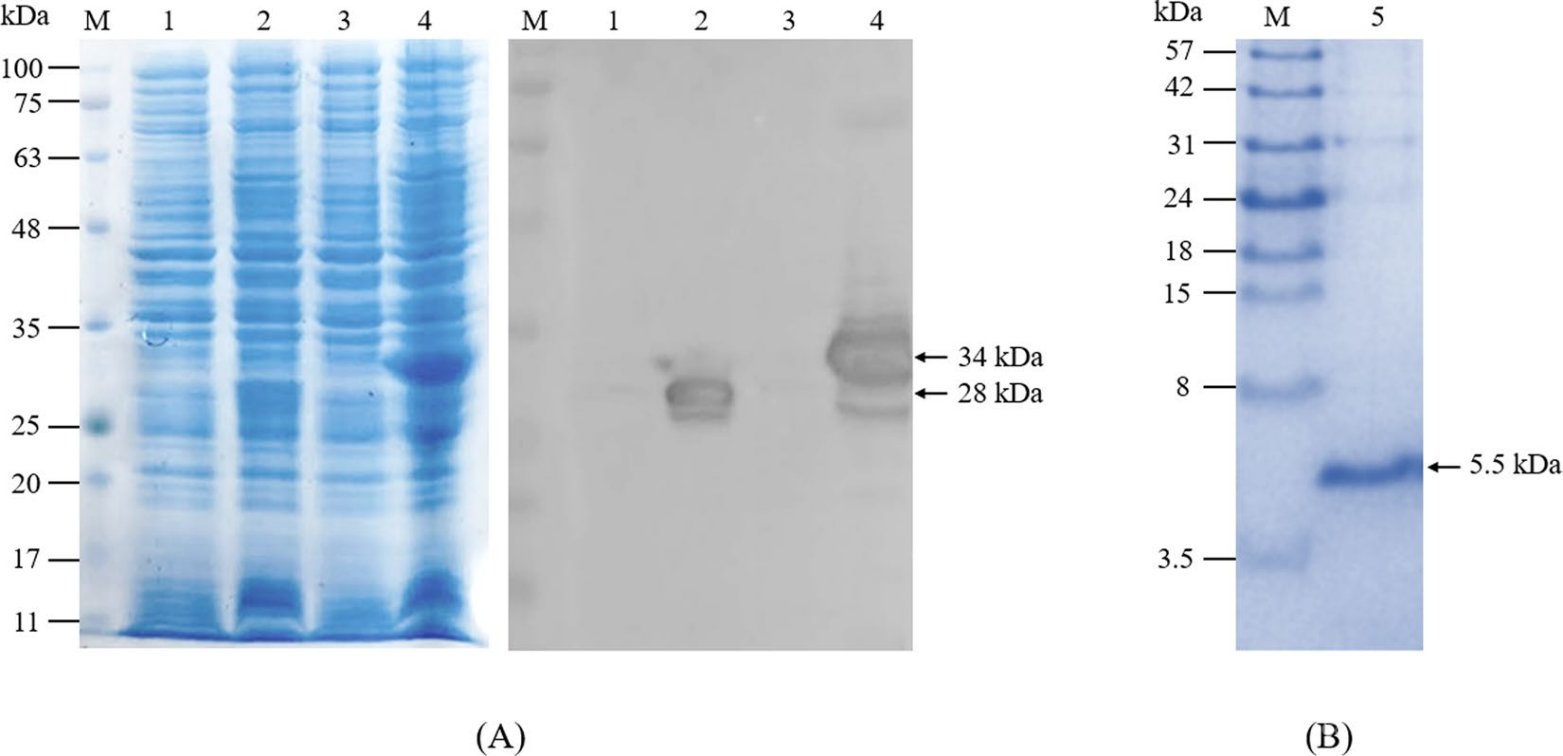
The SDS-PAGE and Western blot analysis of javanicin-intein fusion protein. The *E. coli* origami 2 (DE3), harboring either a pTXB-1 vector control or a pTXB-1-javanicin recombinant plasmid, were induced for protein expression and analyzed by SDS-PAGE. Western blot analysis was employed to confirm javanicin-intein fusion protein expression (**A**). After purification and peptide cleavage, the recombinant javanicin with a molecular weight of approximately 5.5 kDa was determined by 16.5% Tricine SDS-PAGE gel (**B**). Lane M, standard protein marker; Lane 1–2, pTXB-1 transformants before and after IPTG induction; Lane 3–4, pTXB1-javanicin transformants before and after IPTG induction; Lane 5, the 5.5 kDa of purified javanicin after chitin affinity purification.

A single colony of *E. coli* origami 2 (DE3) carrying pTXB-1-Javanicin plasmids was cultured in an LB medium supplemented with antibiotics. After induction, the bacteria were harvested, lysed and determined through a sodium dodecyl sulphate-polyacrylamide gel electrophoresis (SDS-PAGE) analysis. The results revealed that javanicin-intein fusion proteins were expressed mostly in a soluble form with a molecular weight of approximately 34 kDa while the intein-CBD fusion tag in plasmid control was around 28 kDa. Western blot analysis using anti-CBD monoclonal antibodies affirmed an accordance with SDS-PAGE analysis thereby representing a visible protein band of 34 kDa (Fig. 3A). After purification and peptide cleavage, the purified javanicin was analyzed by a tricine SDS-PAGE. The results showed a single band on the gel with a molecular weight of approximately 5.5 kDa as analyzed by Image J software (Fig. 3B), that appears to align with a mass of 5,689.42 Da, which was predicted by the peptide mass software. In addition, the peptide sequence of the purified javanicin was also confirmed by matrix assisted laser desorption ionization-time of flight mass spectrometry (MALDI-TOF MS) (Fig. S1). Isopropyl-1-thio-beta-D-galactopyranoside (IPTG) concentrations, times and temperatures were optimized to maximize fusion protein expression. Image J software was applied for a prediction of the expressed protein. After induction times and temperatures were varied to find the most desirable conditions, the results indicated that the optimal condition for IPTG induction was 0.1 mM, time and temperature was determined to be 6 h after induction at 25 °C respectively (Fig. S2). The estimated target protein expression under optimal conditions was approximately 28% of the total amount of proteins. The recovery yield of purified javanicin was 2.5–3.5 mg/L of cultured bacteria with a purity of over 90%.

### Antimicrobial activity of recombinant javanicin

Standard broth microdilution method was performed to determine the antimicrobial abilities of the recombinant javanicin proteins. The results implied that the recombinant peptides exhibited potent antifungal actions with both yeasts (drug-sensitive and drug-resistant *C. albicans*) and mold (*T. rubrum*) (Fig. S3). Antibacterial activity was unable to be observed. The minimum inhibitory concentration (MIC) value for recombinant javanicin peptides against the microorganisms tested is summarized in Table 1.

**Table 1:** The MIC and MMC value of recombinant javanicin against microbial pathogens.

Microorganisms	MIC (µg/ml)	MMC (µg/ml)
*E. coli* ATCC 25922	>100	ND
*S. aureus* ATCC 25923	>100	ND
*C. albicans* ATCC 90028	100^a^, 50^b^	100
Fluconazole-resistant *C. albicans*	100^a^, 50^b^	100
*C. neoformans*	25^a^	25
*T. rubrum*	25^a^, 12.5^b^	50

^a^MIC_90_; The lowest concentration of antimicrobial agent that prevents any discernible growth

^b^MIC_50_; The lowest concentration of antimicrobial agent that shows prominent (~50%) decrease in turbidity

MMC; The lowest level of antimicrobial agent resulting in microbial death.

### Cytotoxic effects of recombinant javanicin against human breast cancer cell lines

**Figure 4:**
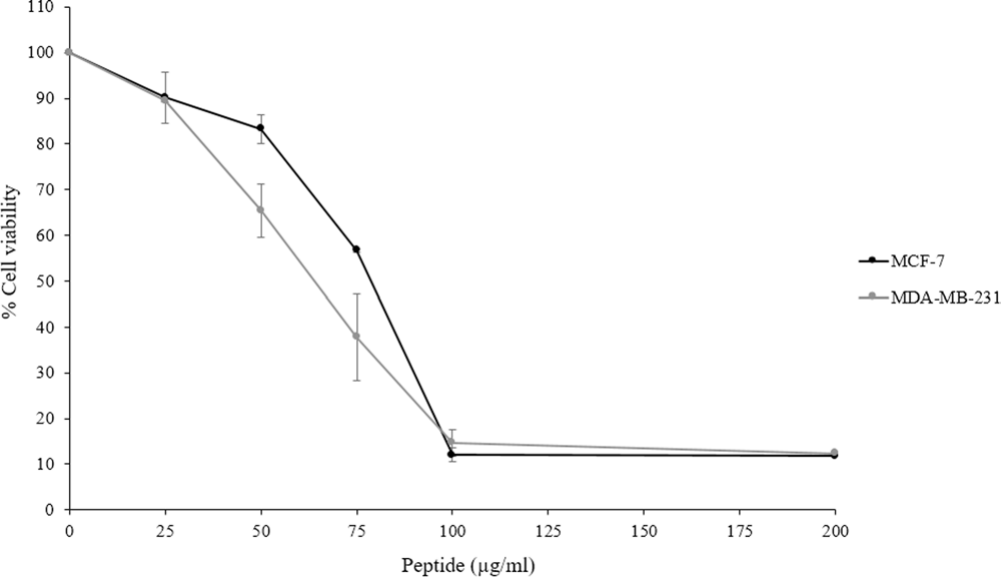
The cytotoxic activity of recombinant javanicin against MCF-7 and MDA-MB-231 cell lines. Various concentrations of recombinant javanicin were examined for determination of anti-proliferative activity against human breast cancer cell lines, MCF-7 & MDA-MB-231. The 50% growth inhibitory concentration (IC_50_) of peptide to each immortalized cell was calculated. The experiment was done in thrice. Error bars indicated standard deviations.

The cytotoxic effect of recombinant javanicin was demonstrated with the human breast cancer cell lines, MCF-7 and MDA-MB-231, using a 3-(4,5-dimethylthiazol-2-yl)-2,5-diphenyl tetrazolium bromide (MTT) assay. The anticancer drug doxorubicin was used as a positive control in this study (Fig. S4). The results showed that javanicin exhibited potent cytotoxic activity against both cancer cell lines in a dose-dependent manner. A 50% growth inhibitory concentration (IC_50_) of recombinant javanicin ranged from 75–85 µg/ml for MCF-7 and 60–85 µg/ml for MDA-MB-231, respectively. The rate of cancer cell deaths over 85% was observed when 100 µg/ml of peptides were examined (Fig. 4).

### Toxicity determination for recombinant javanicin using red blood cell hemolytic assay

**Figure 5:**
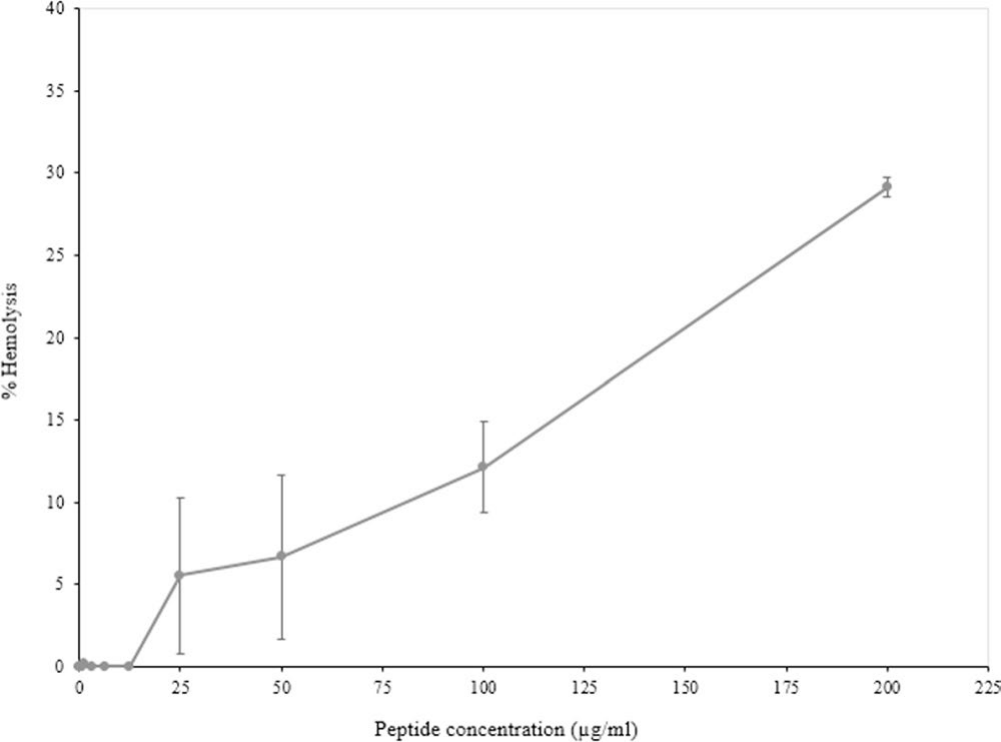
The red blood cell hemolytic assay for *in vitro* toxicity determination of recombinant javanicin. The toxicity determination of recombinant javanicin was performed using red blood cell hemolytic assay. The percent hemolysis versus peptide concentrations were plotted. The peptide suspension buffer and 1% triton X-100 were employed as 0% and 100% hemolysis, respectively. The experiment was done in triplicates. Error bars indicated standard deviations.

The toxicity of recombinant javanicin was analyzed with a red blood cell hemolytic assay. The results indicated that approximately 10% of human blood cells were hemolyzed when a 100 µg/ml peptide concentration was tested. Furthermore, the toxicity against red blood cells increased in a dose-dependent manner to approximately 25% when the peptide concentrations were doubled. The toxicity determination of javanicin is shown in Fig. 5.

## Discussion

The increased appearance of drug-resistant pathogens and cancers has become a major health issue, thus effective treatments for dealing with these hardy diseases are urgently required. Due to the lack of knowledge about the antimicrobial drug pharmacokinetics in cancer patients and the adverse effects of antimicrobial and anticancer drug interactions, the treatment of cancer patients infected with MDR pathogens is known to be quite complicated and represents a major clinical challenge for the medical industry^20^. AMPs with dual antimicrobial and anticancer activities are being considered to solve these problems. During the last decade the list of new AMPs discovered in various types of organisms has been increasing each year^21^. Among these organisms, plants are widely recognized as a valuable source of natural AMPs. Based on structure, activity and cysteine motifs, plant AMPs were classified into at least five families including thionins, knottin, havein-like peptides, snakins and defensins^19^. Among this list the latter is the most relevant and the largest family providing potent and broad-spectrum antimicrobial defenses against microbes and their resistant strains. In addition, some plant defensins are capable of acting against non-contagious diseases including cancer cells, insecticidal properties have also been reported^22,23^. Hence, plant defensins are attracting attention in the development of modern antibiotics to cure MDR infections and many types of cancer.

Herein, we demonstrated the isolation of a defensin gene from the seeds of five tropical plants in the legumes family using 3′ RACE amplification and cDNA cloning. The nucleotides and deduced amino acid sequences of newly discovered plant defensins were identified and recorded into the GenBank database. With consideration to the transcribed mRNA, it was noticed that nucleotide sequence encoding for signal and mature peptides from each plant defensin were highly conserved, nevertheless, the variation of both length and nucleotide sequence of a 3′ untranslated sequence in some plant defensins were observed. This data was in alignment with previous reports that plant defensins are expressed from multigene families^24^. In addition, Thomma & Broekaert also demonstrated that the expression levels of each type of *Arabidopsis thaliana* defensin is dependent on tissue-specifications^24^.

*In silico* characterization of these special plant defensins indicated that they were highly conserved with Fabaceae defensins comprising of a 4-disulfide bond formation of eight cysteine residues at C_1_–C_8_, C_2_–C_5_, C_3_–C_6_ and C_4_–C_7_. The presence of conserved cysteine-rich residues allowed the peptides to maintain their secondary structure and activity. The secondary structures of other plant defensins have previously been published^19^. To show the evolutionary conservation and relationship of identified plant defensins, multiple sequence alignments and phylogenetic trees were determined and compared with other known defensins, the results of both multiple sequence alignments and phylogenetic analyses indicated that these plant defensins were highly conserved and clustered in the maize and soybean clade of the Fabaceae family and Papilionoideae subfamily with eight conserved cysteine residues as previously reported^25^.

AMP predictions using three different algorithms, including SVM, RF and DA, arose from several parameters including amino acid composition, physiochemical properties and secondary structure propensities which could be calculated based on their size, net charge (at physiological pH), pI and hydrophobicity^26,27^. The sequences of our plant defensins showed high scores of AMP prediction and represented physicochemical properties corresponding to the previously reported defensins^28–30^.

Based on the current understanding of plant defensins activities and characteristics, large amounts of bioactive peptides with a high purity are provided. Although chemical peptide synthesis could be cost effective with several types of AMPs, the cost for large scale production of long peptides is a major limitation of this process^31^. Based on its fast growth and low cost, a recombinant AMP expression in a bacterial system was utilized for this study^32^. *S. javanica* defensin was selected as a candidate peptide to determine its antimicrobial and anticancer activities. Genes encoded for the javanicin mature peptide, in addition to methionine (ATG), were inserted at the N-terminus of an intein-CBD sequence of pTXB-1 plasmid and expressed as a fusion protein in an *E. coli* Origami 2 (DE3) strain. The fusion partner utilized for recombinant AMP expression was not only intended to avoid the proteolytic degradation of the peptide and lower the natural toxicity of the AMP against the engineering host, but also to increase the yield of protein production and facilitate AMP purification and processing^33^. After expression, a soluble form of javanicin-intein fusion protein was obtained with a molecular mass of approximately 34 kDa, and was estimated to be 28% of the total bacterial protein amount. With some experience in heterologous protein expression, it was realized that the eukaryotic gene expressed in prokaryotic cells is always produced in an aggregated form. This is due to the disability of post-translation modification or a refolding of the target protein^34^. Therefore, the lack of thioredoxin reductase and glutathione reductase in the commercial Origami strain used in this study enhanced disulfide bond formation of cysteine-rich javanicin and possibly promoted correct refolding^35^. After thiol induced intein cleavage, the purified javanicin with a molecular weight of 5.5 kDa was collected. The recovery yield of purified peptide was approximately 2.5–3.5 mg/L of cultured bacteria with over 90% purity. The recovery yield of other recombinant plant defensins have previously been reported with a range of 0.63–50 mg/L, dependent on the expression system^36,37^. Therefore, it was revealed that PDC1, a corn defensin that was expressed from a bacterial expression system, exerted less antifungal activity than a yeast system^38^. The post-translation modification and refolding in eukaryotic cell systems may be associated with the enhancement of antifungal activity. Antimicrobial activity determination was performed in both bacteria and fungi and the results demonstrated that recombinant javanicin showed a potent antifungal action against human pathogenic yeasts and molds. In addition, the fungicidal activity of the peptide against fluconazole-resistant *C. albicans* was also observed. Because of their effectiveness and low cost, azole antifungal drugs are the most commonly used in the treatment of *Candida* infections. Therefore, the hepato- and nephrotoxicity induced by these drugs are some of the major limitations in the treatment of fungal infections. Currently, the occurrence of azole resistant *C. albicans* is increasing globally^39^ therefore, recombinant the javanicin produced in this study might be clinically useful in the treatment of drug resistant fungal infections. Plant defensins are nowadays classified into four groups depending on their ability to inhibit bacterial and/or fungal growth as well as their effects on fungal morphology following the treatment. Group I is comprised of the defensins that inhibit both bacteria and fungi and cause the distortion of hyphal branching whereas group II defensins had an ability to inhibit only fungal pathogens without inducing hyphal branching. Group III defensins caused the specific inhibition of bacteria while group IV defensins represent both antibacterial and antifungal activity without inducing hyphal branching^40^. In the present study, we propose that recombinant javanicin has a behavior resembling group II defensins. A variety of fungal target molecules, that plant defensins can block and impair the function of, have previously been identified such as the cyclin F protein related to cell cycle control in fungal nuclei^41^, ergosterol and glucosylceramide in fungal membranes^42^. Therefore, the additional information about how javanicin affects fungal growth inhibition and causes fungal cell death is being investigated in our laboratory. Initially, the analysis of scanning electron microscope results indicated that recombinant javanicin inhibits the growth of pathogenic yeast through non-membrane disruption when sub-MIC concentrations were incubated with *C. albicans in vitro* (Data not shown).

Recombinant javanicin also exerted anti-proliferative activity against the human breast cancer cell lines, MCF-7 and MDA-MB-231, with little difference in IC_50_ values between both cell types, this was similar to the MIC of antifungal effects. As previously described, almost all AMPs displayed antimicrobial activity against microbial pathogens^43^. Few AMPs have the ability of dual action when it comes to antimicrobial and cytotoxic activities toward the cancer cells. Over the past ten years the modes of action of AMPs against microorganisms have been well documented, the most common mode of action on microbes being the disruption of microbial membranes. Although the exact molecular effect of AMPs have on cancer cells is not yet fully understood, it appears that AMPs anticancer effect is similar to that of its action against microbial pathogens^44^. Based on the molecular biology of membrane phospholipids, the negatively charged phosphatidylserine (PS) is generally deposited on the plasma membranes’ inner leaflet of normal cells. Therefore, the loss of PS symmetry between the inner and outer membrane leaflets of cancer cells leads to the presence of a negatively charged side on the outer membrane. This situation promotes peptides binding through electrostatic interaction and ultimately results in cancer cell death via the increase in membrane permeability and/or targeting of sub-cellular organelles^45^.

Considering the phenotype of human breast cancer cells used in this study, a few physiological characteristics of the MDA-MB-231, which are distinctly different from MCF-7, should be considered including the lack of oestrogen receptors and progesterone receptor expressions^46^. Consequently, the MCF-7 cell line is usually applied for the study of hormonal therapy whereas the MDA-MB-231 is more compatible for chemotherapeutic models. In our data, a similar concentration of peptides exhibited anti-proliferative activities against both cancer cell types. Hence, neither the presence of oestrogen receptor nor progesterone receptor exposure to the cancer cells surface may be the initial target for recombinant javanicin to bind to, which would in turn cause cancer cell death. The cell viability after peptide treated was initially performed using acridine orange/ propidium iodide (AO/PI) staining and visualized under the fluorescence microscope. After treated with recombinant javanicin, the number of dead cells with red fluorescence intensity are increasing in dose-dependent manner (Fig. S5). However, the morphological changes inside the cells is unclear. To find out the mechanism for cancer cell death occurring from javanicins activities, FITC-labelled annexin V/PI double staining was used, which allowed for apoptotic cell detection. This was performed with MCF-7 cells treated with either sub-IC_50_ or IC_50_ concentrations of the peptide and analyzed through a flow cytometer. The anticancer drug doxorubicin was used as a positive control in this study. The preliminary findings indicate that javanicin is capable of inducing cancer cell death through apoptosis in a dose-dependent manner (Fig. S6). Therefore, the molecular target of recombinant javanicin involved in cancer cell death is being further investigated in our laboratory. Recently, the mode of action of cationic antimicrobial peptides (CAPs) against MCF-7 cell line was demonstrated^47^. It was discovered that the CAPs initially interact with negatively charged PS and made their way into MCF-7 cells through clathrin-independent macropinocytosis and finally caused cancer cell death by inducing nuclear damage.

A red blood cell hemolytic assay was performed to determine the *in vitro* toxicity of recombinant javanicin. The results indicated an increase in undesired hemolysis in a dose-dependent manner while the higher concentrations of recombinant javanicin were also examined. Similar findings were also found in another plant defensin in a previous study, this defensin is known as NoD173, a plant defensin isolated from *N. Occidentalis*^48^. Several crucial physiochemical properties that are associated with the antimicrobial activities and target specificity of AMPs include the peptides size, charge, hydrophobicity, amphipathicity and solubility^49,50^. When taking these factors into account, changing these features through amino acid substitution may improve the activity and target specificity of recombinant javanicin.

In summary, the defensin genes from five types of legume seeds were successfully isolated and identified by 3′ RACE. The *S. javanicas’* mature defensin gene was fused with intein-CBD fusion tags and expressed as a fusion protein in *E. coli*. The recombinant javanicin showed potent antifungal and anticancer activities. For this reason, the actual targets of javanicin in fungal and cancer cells destruction are under further investigation. This study provides a promising platform for the study of legume defensins and a model for AMP expression in *E. coli* that enables high levels of peptide expression and facilitates the one-step purification of soluble and active peptides.

## Methods

### International biosafety committee approval

In this study, the research work associated with recombinant DNA technology was approved by the International Biosafety Committee (IBC), Chiang Mai University. The approval number is CMU IBC A-0561007.

### Plant materials

Legume seeds were purchased from either Doikham Royal Project or from local markets in the province of Chiang Mai, Thailand. Seeds were then cleaned, weighed and ground in liquid nitrogen. The resulting powder made from the seeds was immediately stored at −80 °C until undergoing nucleic acid extraction.

### Microorganisms and cell lines

Standard strains of *E. coli* ATCC 25922, *S. aureus* ATCC 25923, *C. albicans* ATCC 90028, clinical isolates of fluconazole-resistant *C. albicans*, *C. neoformans* and the clinical isolate mold strain *T. rubrum* were provided by the Division of Clinical Microbiology, Department of Medical Technology. Human breast cancer cell lines, MCF-7 and MDA-MB-231, were provided by the Department of Radiologic Technology, Faculty of Associated Medical Sciences, Chiang Mai University.

### RNA extraction and cDNA synthesis

Total RNA was extracted from 200 mg of seed powder following the established protocol^51^. Immature seeds were initially homogenized in liquid nitrogen using a sterile mortar and pestle. After the addition of an SDS/Trizol solution, an aqueous phase containing RNA was collected through high speed centrifugation. The RNA was precipitated using ethyl alcohol, washed and dissolved in sterile double-distilled water (ddH_2_O). A reverse transcription reaction was prepared by mixing 2000 ng of total RNA, 10 mM oligo dT primer and commercial reagents (Invitrogen, Carlsbad, CA). The reaction was incubated at 50 °C for 60 min. The cDNA product was stored at −20 °C until PCR amplification.

### Defensin encoding gene amplification by 3′ RACE, cDNA cloning and direct sequencing

A defensin encoding gene was amplified using a 3′ RACE technique. A full-length sequence was carried out using degenerate primers that were designed using nucleotide sequences from a Fabaceae defensin pre-peptide gene. A PCR reaction was achieved in a total volume of 50 µl containing 10 × reaction buffer, 2 mM dNTP, 25 mM MgSO_4,_ 10 µM DEF1-Forward primer, 10 µM Oligo dT primer and 1 U of KOD-plus-Neo Taq DNA polymerase enzyme (Toyobo, Osaka, Japan). The nucleotide sequence of primers used are shown in Table 2. The reaction was initially conducted by using a pre-denaturation program at 94 °C for 2 min, followed by 45 cycles of 98 °C for 10 sec, 50 °C for 30 sec, 72 °C for 30 sec, with a final extension at 72 °C for 7 min. The amplified product was used as a template for subsequent amplification using a DEF2-Forward primer with the conditions described above. The amplified product from each plant (approximately 250–450 bp) was cleaned and cloned into a pJET 1.2/blunt vector (Thermo Fisher scientific, Waltham, MA) and transformed into *E. coli* TOP 10 F according to a standard heat shock technique^52^. From each plant, ten positive clones harboring plasmids pre-screened by a colony-PCR were extracted from the overnight culture using NucleoSpin^®^ Plasmid purification (Macherey-Nagel, Duren, Germany) and were then sequenced using vector specific primers.

**Table 2:** The nucleotide sequence of primers used in this study.

Primer name	Nucleotide sequence (5′-3′)*
DEF 1	GGCCATATGGASAAGAAATCM
DEF 2	TGCTTCCTCTTCCTYGTTCT
P1	TACGTACCGCGGTCCGTGCTTCACTACCGCCAGCTGTGAC GATCACTGCAAAAACAAA
P2	CAACAGCGAAAATCGTCCCGGCAGCGGCCACGAACCAGAT GTTCTTTGTTTTTGCAGTG
P3	GGTGGT**CATATG**CGTACCTGTGAAAACCTGGCGGATACGT ACCGCGGTCC
P4	GGTGGT*TGCTCTTC*CGCAACAGTTACGAGTGCACCAACAG CGAAAATCG

^*^Bolds and italics are *Nde*I and *Sap*I restriction sites, respectively.

### Bioinformatic analysis

Various bioinformatic tools were used for an *in silico* characterization of plant defensins including the ExPASy translation tool, ExPASy Compute pI/Mw tool, SignalP 4.1, Clustal X 2.1 & GeneDoc 2.7 public software for multiple sequence alignment, MEGA version 6 for phylogenetic study and the Collection of Anti-Microbial Peptides (CAMP_R3_) database for prediction of antimicrobial peptides probability.

### Construction of javanicin gene

Javanicin, a novel defensin obtained from *S. javanica* seeds, was selected as a candidate sequence for recombinant peptide expression and characterization in this study. DNA fragment encoding for javanicin was generated by using SOE-PCR. The inner fragment was initially amplified through P1 and P2 primers and the amplified product was subsequently amplified for full length construction using P3 and P4 primers. The nucleotide sequence of primers is shown in Table 2. PCR profiles for both amplification steps were as follows: 30 cycles at 94 °C for 30 sec, 54 °C for 30 sec and 72 °C for 30 sec, followed by a final elongation step at 72 °C for 5 min. The amplified products were double digested with *Nde*I and *Sap*I restriction enzymes, purified, ligated into linearized pTXB1 expression plasmid (New England Biolabs Inc., USA) and transformed into *E. coli* Origami 2 (DE3). The vector harboring a javanicin-intein-CBD was screened by a colony-PCR using vector primers and the integrity of in-frame fusion was verified using DNA sequencing.

### Expression of recombinant javanicin in *E. coli*

*Escherichia coli* Origami 2 (DE3) cells harboring javanicin-intein-CBD plasmid were freshly grown in an LB medium supplemented with antibiotics until the optical density reached 0.6. The IPTG, a final concentration of 0.1 mM was added and further incubated by shaking the solution at 25 °C for 6 h. The bacterial cells were harvested by centrifugation at 4000 g, 4 °C for 10 min. The recombinant javanicin-intein-CBD fusion protein was detected by SDS-PAGE.

### Optimization of recombinant javanicin expression

To optimize the expression of recombinant javanicin in bacteria, the concentrations of IPTG, times and temperatures were varied. The IPTG was added to a final concentration of 0.1, 1 and 10 mM and the bacteria was further cultured at 25 °C for 24 h. After that, the bacterial cells were harvested and analyzed by a 12% SDS-PAGE gel. Times and temperatures after IPTG induction also varied from 0–24 h and 20–37 °C, respectively. Bacteria was collected and the recombinant javanicin-intein-CBD fusion protein was further determined by SDS-PAGE analysis. A band of recombinant protein was estimated with the use of Image J 1.52a public software.

### Javanicin fusion protein expression confirmed by western blot analysis

Western blot analysis was undertaken to determine the javanicin-intein-CBD fusion protein expression. The protocol was followed by the previous report^53^. The commercial anti-CBD monoclonal antibody (New England Biolabs, Ipswich, MA) and horseradish peroxidase (HRP)-conjugated goat anti-mouse Ig G antibody (Dako Cytomation, Glostrup, Denmark) were used as primary and secondary antibodies in this study.

### Affinity purification and peptide cleavage of recombinant javanicin

The affinity purification and peptide cleavage of the recombinant protein was performed as previously described^53^. Briefly, bacterial pellets were lysed using a commercial B-PER bacterial protein extraction reagent (Thermo Fisher scientific, Waltham, MA). The supernatant was collected via centrifugation at 4 °C, 10000 g for 20 min. The clarified extract, containing the javanicin-intein-CBD fusion proteins, was applied into a chitin affinity column (New England Biolabs, Ipswich, MA). The following steps were done according to the manufacturer’s instructions. The excised peptides were collected by restoring buffer flow through the column, repetitively concentrated using 3000 Da ultracentrifugation (Amicon Ultra Centrifugal Filters; Merck Millipore, Burlington, MA) and subsequently dialyzed in PBS, pH 7.4 at 4 °C overnight. The peptide concentrations were ascertained, aliquoted and kept at −80 °C until determination.

### Antimicrobial activity testing

To determine the antimicrobial effect of recombinant javanicin, standard strains of *E. coli*, *S. aureus* and *C. albicans* and clinical strains of fluconazole-resistant *C. albicans*, *C. neoformans* and *T. rubrum* were selected to be the candidate microorganisms in this study. Standard broth microdilution was employed to determine the MIC according to the Clinical and Laboratory Standard Institute (CLSI) guidelines, M07-A9 for bacteria (2012) and M27-A3 for yeast (2008). Briefly, the bacteria and yeast were grown in trypticase soy broth (TSB) at 37 °C then agitated at 200 rpm overnight. The mold, *T. rubrum*, was inoculated in potato dextrose agar (PDA) and cultured for 5–7 days at 25 °C. The inoculum of yeast and mold spores was adjusted using a hemocytometer. For the bacteria, the cell density was optically adjusted with McFarland standard turbidity No. 0.5. Approximately 5 × 10^5^ CFU ml^−1^ of bacteria and 5 × 10^3^ CFU ml^−1^ of yeast and fungal spores were added into a diluted peptide solution (0.4–100 µg/ml) for a final volume of 100 µl. Following the incubation, plates were determined after 24, 48 and 72 h. The experiments were separately performed in triplicate and the average MIC value was measured. To determine the minimum microbicidal concentration (MMC), twenty microliters from the clear wells on 96-well plates were collected and plated on agar. The plates were then incubated at 37 °C until development was seen in the growth control subculture. The lowest drug concentration that showed zero growth of microorganisms was determined to be the MMC value.

### Cytotoxic activity of recombinant javanicin

The cytotoxic activity of recombinant javanicin was evaluated using a MTT assay^54^. All reagents and cell culture medias were purchased from Gibco Company (Dublin, Ireland). After the incubation of either MCF-7 or MDA-MB-231 cell lines with diluted peptide (0–100 µg/ml), the MTT solution (0.5 mg/ml) was added and further incubated at 37 °C for 4 h. The color developed was determined by measuring the absorbance at 555 and 800 nm using a microplate reader (BioTek^TM^ Eon^TM^ microplate reader). All assays were carried out three times.

### Red cell hemolytic assay

A red blood cell hemolytic assay was performed to determine the toxicity of the recombinant javanicin. The protocol has previously been described elsewhere^33^.

## Supplementary information

Supplementary information
