# Supplementary material for: A novel recombinant javanicin with dual antifungal and anti-proliferative activities
Source: Sci Rep. 2019 Dec 5;9:18417. doi: 10.1038/s41598-019-55044-7 (PMC6895105; doi:10.1038/s41598-019-55044-7)
Supplement: Supplementary file 1 — Supplementary information [file 41598_2019_55044_MOESM1_ESM.docx]

Supplemented data

**A novel recombinant javanicin with dual antifungal**

**and anti-proliferative activities**

Santhasiri Orrapin^1^, Amornrat Intorasoot^2^, Sittiruk Roytrakul^3^, Nathupakorn Dechsupa^4^, Jiraporn Kantapan^4^, Yanika Onphat^1^, Chutima Srimek^1^, Chayada Sitthidet Tharinjaroen^1,5^, Usanee Anukool^1,5^, Bordin Butr-Indr^1,5^, Ponrut Phunpae^1,5^, Sorasak Intorasoot^1,5,#^


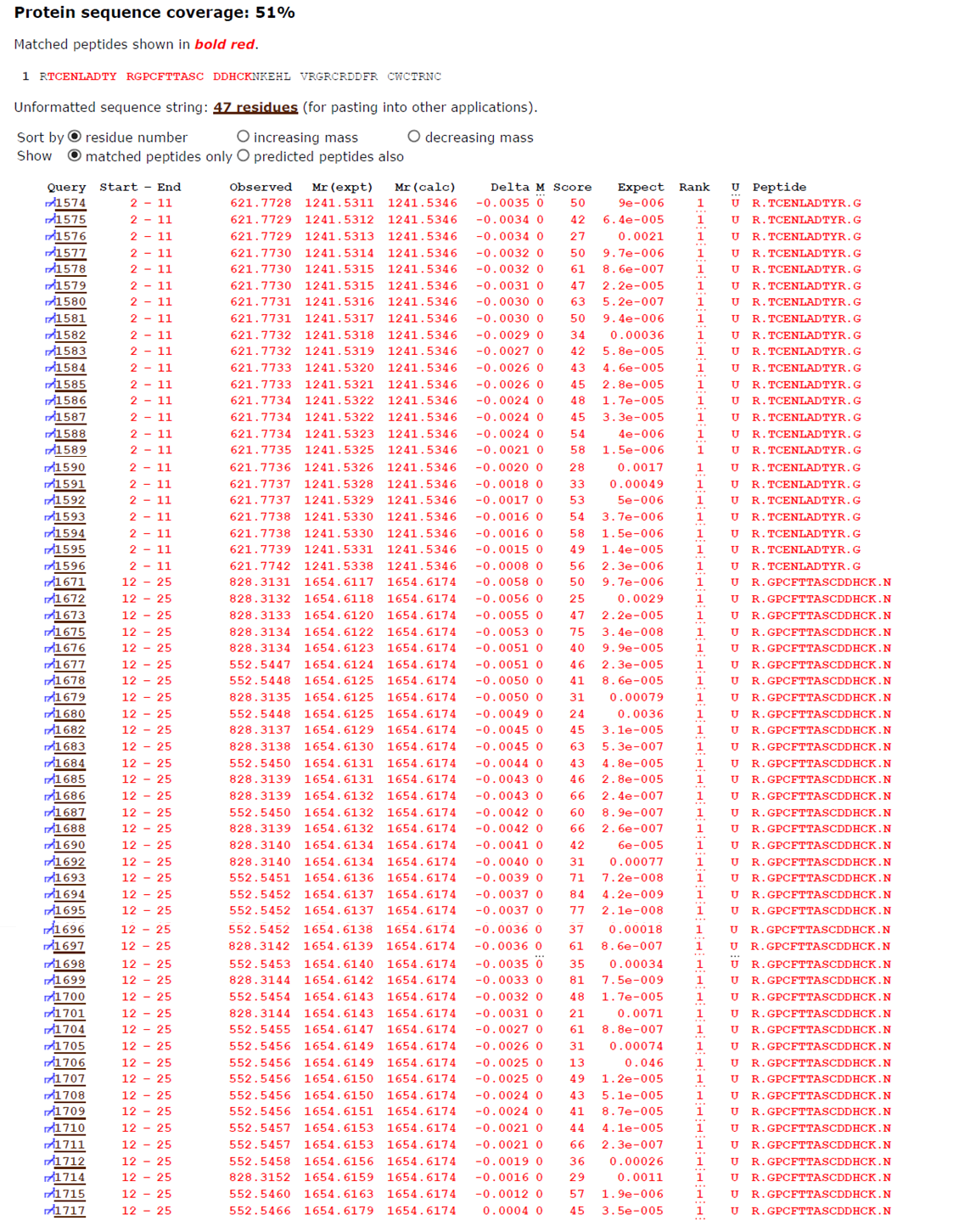


Fig. S1 The analysis of peptide sequence of purified javanicin by MALDI-TOF Mass spectrometer

The purified javanicin is examined using MALDI-TOF Mass spectrometer and analyzed by the Mascot public software. The 51% protein sequence coverage is indicated to be javanicin.


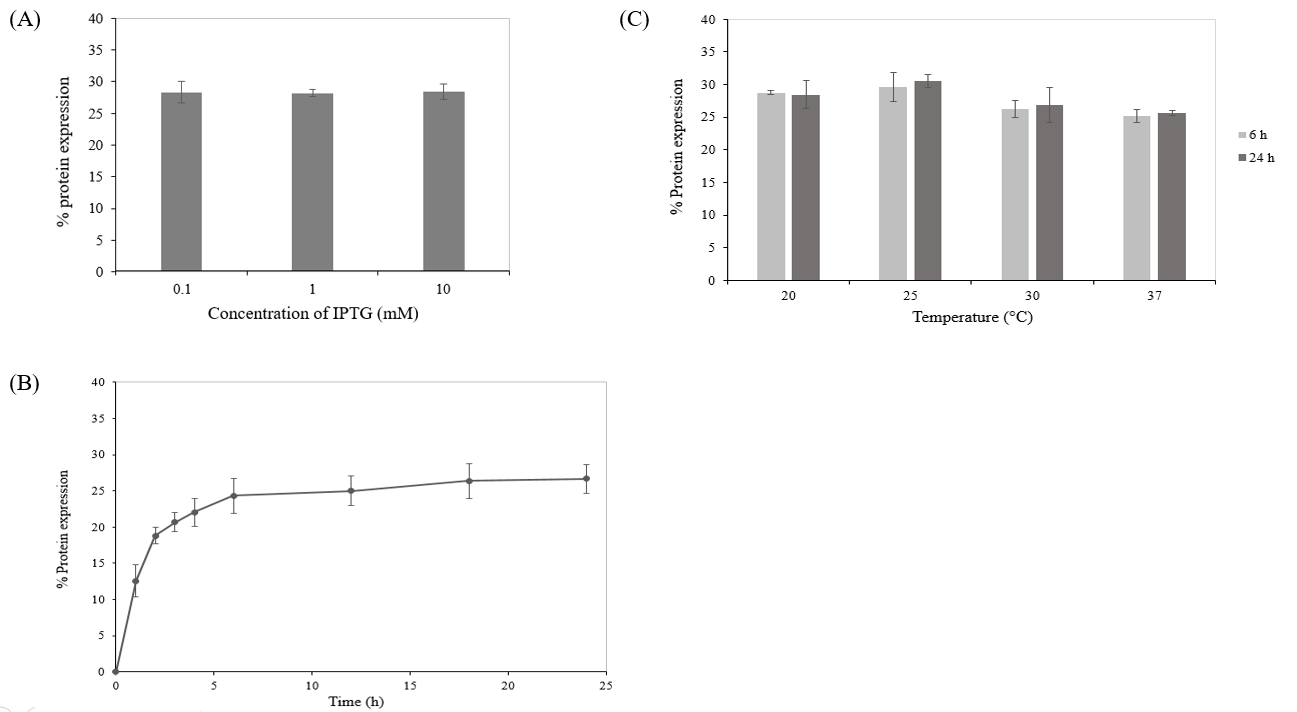


Fig. S2 The expression of javanicin-intein fusion protein in diﬀerent IPTG concentrations, time points after induction and temperatures

The percent of javanicin-intein fusion protein analyzed by SDS-PAGE versus various concentrations of IPTG ranging from 0.1 to 10 mM (A), time points (0–24 h) after 1 mM IPTG induction (B) and temperatures utilized for target protein expression (C) are plotted. The optimal conditions for IPTG induction, time after induction and temperature are 0.1 mM, 6 h and 25 °C, respectively. Each experiment is done in triplicates. Error bars indicate standard deviations.


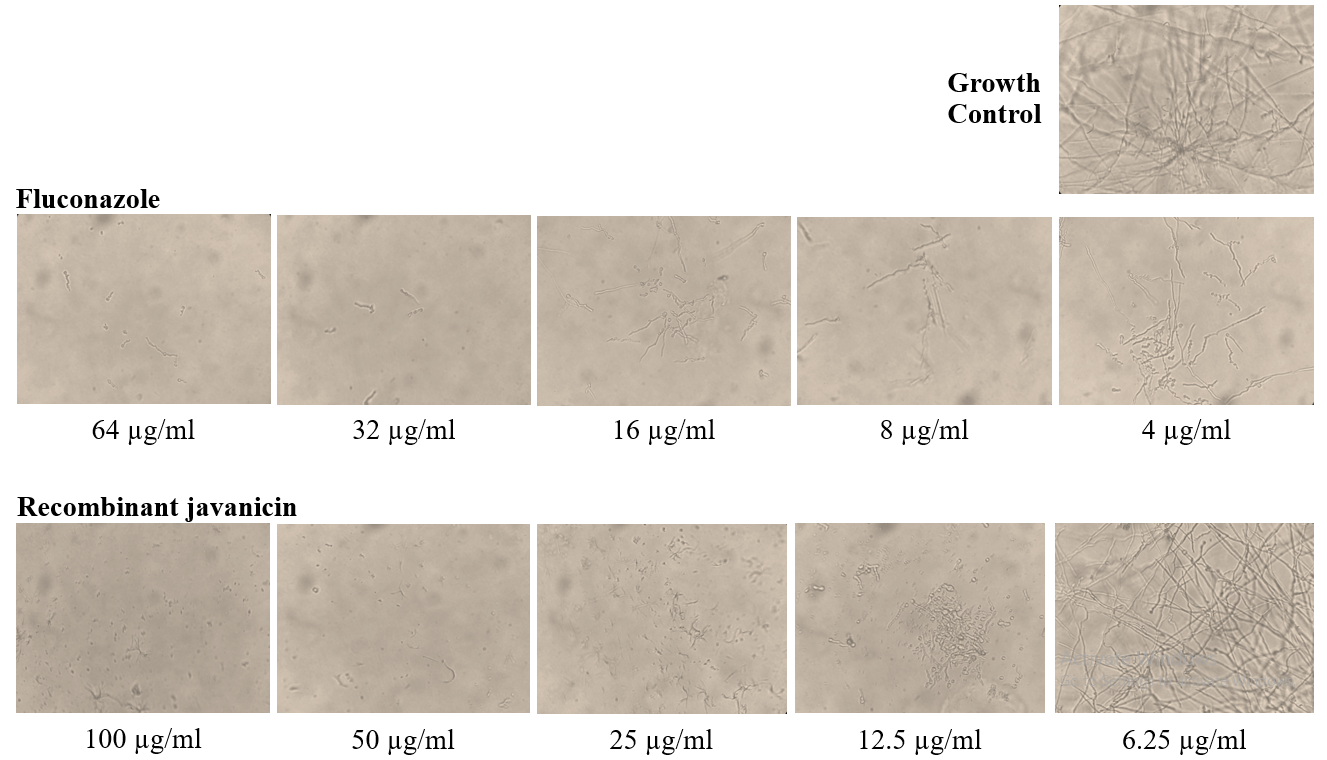


Fig. S3 The broth microdilution assay for antifungal activity determination of recombinant javanicin against *T. rubrum*

The broth microdilution assay is performed for antifungal activity determination of recombinant javanicin against *T. rubrum*. Various concentrations of either javanicin or antifungal drug fluconazole used as positive control are tested with dermatophytic mold and the fungal morphology was observed under the inverted microscope (magnification: 100x).


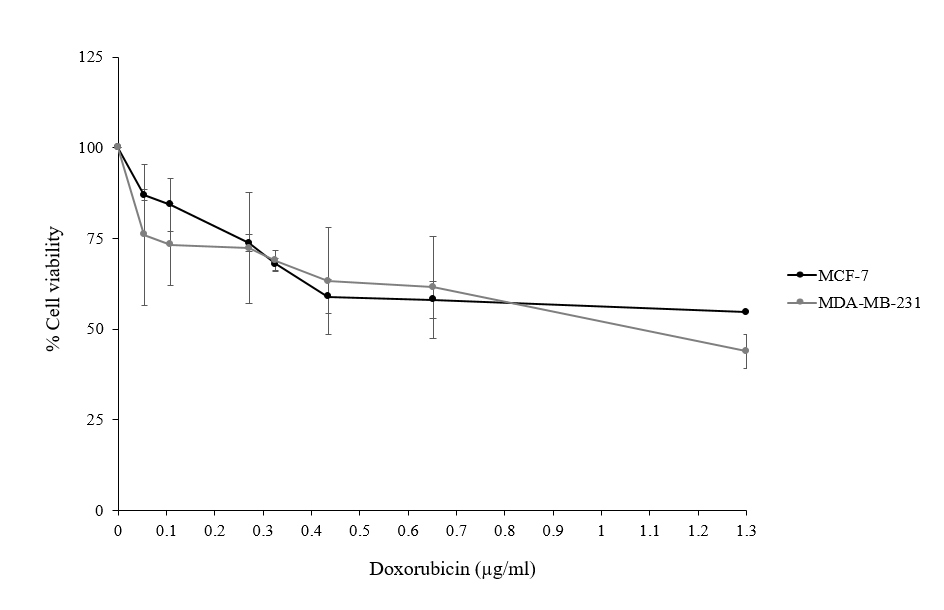


Fig. S4 The cytotoxic activity of doxorubicin against the MCF-7 and MDA-MB-231 cell lines

Several concentrations of anticancer drug doxorubicin used as a positive anti-proliferative activity control in this study are examined against human breast cancer cell lines, MCF-7 & MDA-MB-231. The 50% growth inhibitory concentration (IC_50_) of drug to each immortalized cell was calculated and applied for the next experimentation. The experiment for cytotoxic activity is done in thrice. Error bars indicate standard deviations.


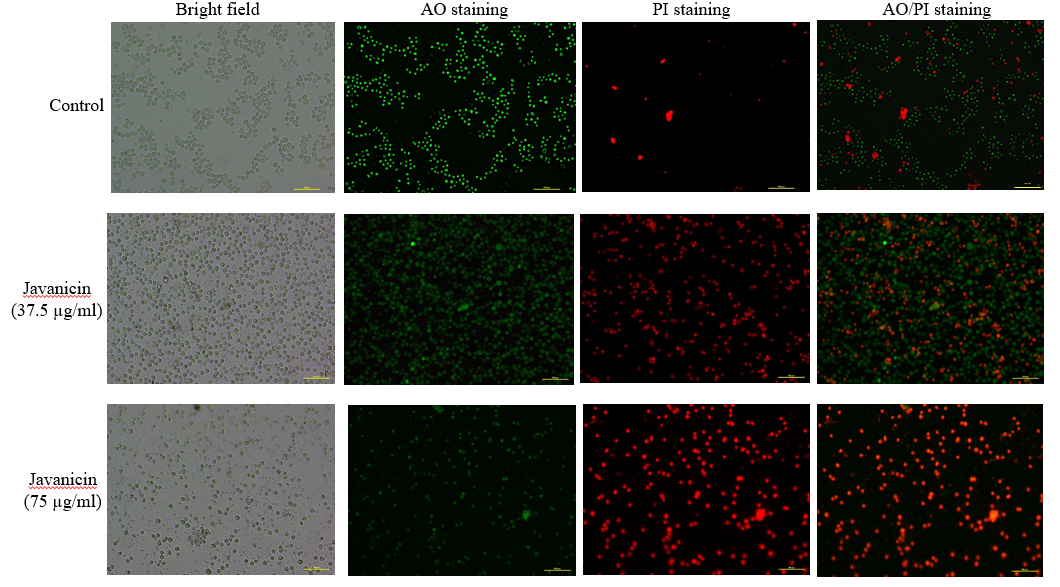


Fig. S5 Fluorescence microscopy for viability study of javanicin treated MCF-7 cells using acridine orange/propidium iodide (AO/PI) double staining

The MCF-7 untreated cells control and cells after treated with sub-IC_50_ and IC_50_ concentration of recombinant javanicin for 48 hr are stained using AO/PI fluorescein dyes.

The untreated control indicated the viable cells are generally stained in green fluorescence by the AO dye. After treated with javanicin, the dead cells, indicated the red fluorescence where PI can be permeated through the damage cell membrane, are observed. Recombinant javanicin induces the MCF-7 cell death in dose-dependent manner. The bar is 100 µm.


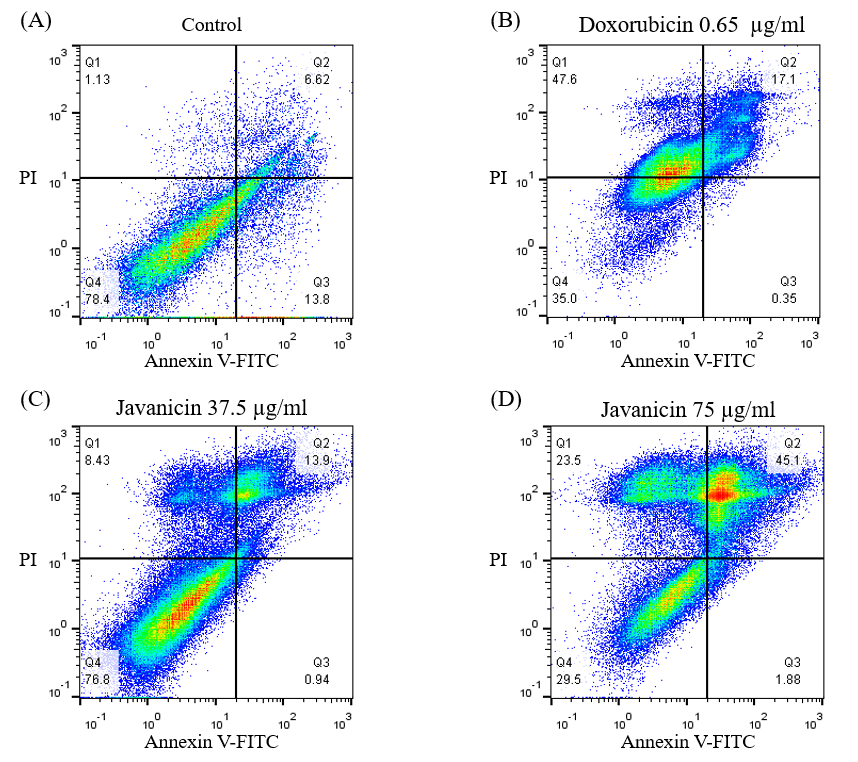


Fig. S6 The annexin V-FITC/PI double staining and flow cytometry analysis for apoptotic cells detection of MCF-7 cells after treated with recombinant javanicin

The MCF-7 cells are treated with either anticancer drug doxorubicin or recombinant javanicin for 48 hr, stained with annexin V-FITC/PI and analyzed by flow cytometer. The results of untreated cell control, cells treated with IC_50_ value of doxorubicin, and cells treated with either sub-IC_50_ (37.5 µg/ml) or IC_50_ (75 µg/ml) value of javanicin are represented in A-D, respectively. In each subfigure, viable cells are indicated in the lower-left quadrant (Q4; Annexin V-FITC/PI: -/-). The early and late apoptosis of cells are presented in the lower-right (Q3; Annexin V-FITC/PI: +/-) and upper-right quadrant (Q2; Annexin V-FITC/PI: +/+), respectively. Cell death via necrosis can be detected in the upper-left quadrant (Q1; Annexin V-FITC/PI: -/+). The percent of cell apoptosis is measured by flow cytometry analysis. The percent of late apoptotic cells are increasing from 13.9% to 45.1% while the peptide concentration is increased from sub-IC_50_ to IC_50_ value.
